# Complex IV subunit 1 defect predicts postoperative survival in hepatocellular carcinoma

**DOI:** 10.3892/ol.2014.1966

**Published:** 2014-03-11

**Authors:** PUO-HSIEN LE, SHIH-CHIANG HUANG, SIEW-NA LIM, CHANG-HUA CHOU, TA-SEN YEH, TSE-CHING CHEN, TZUNG-HAI YEN, MING-YAO SU, CHENG-TANG CHIU, CHAU-TING YEH, WEY-RAN LIN

**Affiliations:** 1 Department of Gastroenterology and Hepatology, Linkou Chang Gung Memorial Hospital, Chang Gung University College of Medicine, Taoyuan 333, Taiwan, R.O.C.; 2 Department of Pathology, Linkou Chang Gung Memorial Hospital, Chang Gung University College of Medicine, Taoyuan 333, Taiwan, R.O.C.; 3 Department of Neurology, Linkou Chang Gung Memorial Hospital, Chang Gung University College of Medicine, Taoyuan 333, Taiwan, R.O.C.; 4 Department of General Surgery, Linkou Chang Gung Memorial Hospital, Chang Gung University College of Medicine, Taoyuan 333, Taiwan, R.O.C.; 5 Department of Nephrology, Linkou Chang Gung Memorial Hospital, Chang Gung University College of Medicine, Taoyuan 333, Taiwan, R.O.C.; 6 Liver Research Center, Linkou Chang Gung Memorial Hospital, Chang Gung University College of Medicine, Taoyuan 333, Taiwan, R.O.C.

**Keywords:** NADH dehydrogenase, cytochrome *b*, cytochrome *c* oxidase, ATP synthase

## Abstract

Mitochondrial oxidative phosphorylation (OXPHOS) is responsible for adenosine triphosphate synthesis and OXPHOS deficiency plays a significant role in tumorigenesis. The defects of mitochondrial-encoded OXPHOS subunits have been found in normal and cirrhotic liver, however their contributions in hepatocellular carcinoma (HCC) are not clear. The present study aimed to examine these defects in resected HCC tissues. In total, 102 human HCC tissues were collected from patients undergoing curative resection, and immunohistochemical staining was performed to assess tissue expression of complex I subunit 6, complex III subunit 3, complex IV subunit 1 (CIV-1) and complex V subunit 6. Cox proportional hazard model analysis was performed, including all clinicopathological factors, to postoperatively estimate the overall survival rate. The results showed that the majority of HCC tissues contained various degrees of expression defects for OXPHOS subunits. Among these, the major CIV-1 defect (expression defect area of >25% of the examined area) (P<0.001) and early distant metastasis (P<0.001) were independently associated with the overall survival rate. Kaplan-Meier analysis also demonstrated that the major CIV-1 defect was significantly associated with a poor overall survival rate (log-rank, P=0.002). The findings in the present study clearly indicate that the major CIV-1 expression defect may serve as an independent negative prognostic factor in HCC patients following curative resection.

## Introduction

Hepatocellular carcinoma (HCC) is the sixth most common solid cancer and the third leading cause of cancer-related mortality worldwide (1). The occurrence of HCC is multi-factorial and it often develops under an established background of chronic liver diseases (2–4). The most outstanding risk factor in Eastern Asia is chronic hepatitis B virus (HBV) infection, while in Japan, Europe and North America, hepatitis C virus (HCV) infection is the notable risk factor, synergistically with alcohol abuse (5). Liver resection is potentially a curative therapy in HCC patients who are within the Milan criteria and have an adequate liver reserve. However, tumor recurrence occurs in >50% of cases within 5 years following surgery, combining true recurrence of the original cancer, which usually arises within the first 2 years, and *de novo* tumor formation (6). Early recurrence is associated with microvascular invasion, poor histological differentiation, satellites and multifocal disease, while late recurrence is mainly dependent on the oncogenic potential of underlying chronic liver disease (7,8). Specific molecules have been identified as factors for predicting postoperative survival. These include proline-directed protein kinase FA, mitogen-activated protein kinase phosphatase-1, vascular endothelial growth factor, proliferating cell nuclear antigen, p53, tissue factor, cytokeratin-19, telomerase activity and interleukin-10 (4,9–16). In patients with HBV-related HCC, the HBV basal core promoter mutation and HBV-DNA level in liver tissues also predict a poor postoperative survival rate (17).

Oxidative phosphorylation (OXPHOS) in mitochondria provides biological energy for intracellular metabolic pathways (18). It is particularly significant in hepatocytes as the liver is one of the most energy-consuming organs. In 1930, Warburg proposed that cancer originated from an irreversible injury to mitochondrial OXPHOS, which forced cancer cells to shift to an energy-generation process through glycolysis despite the presence of aerobic conditions. This condition has been named as the Warburg effect (19). It renders cancer cells capable of surviving and proliferating under adverse conditions. Mitochondrial DNA (mtDNA) is more susceptible to oxidative damage and has a higher mutation rate than nuclear DNA due to a lack of protective histones, limited DNA repair activities and a proximity to the high rate of reactive oxygen species generated in mitochondria (20–22). As such, the accumulation of mtDNA alterations in cancer cells leading to defects in adenosine triphosphate (ATP) generation through the OXPHOS system is consistent with the theory of the Warburg effect. Mutations in mtDNA have been reported in various types of human cancers (23–25). These findings indicate that defects of the OXPHOS complex with mitochondrial-encoded subunits may be a decisive factor in hepatocarcinogenesis.

The OXPHOS process consists of five complexes, which are complex I (NADH: ubiquinone oxidoreductase), complex II (succinate dehydrogenase), complex III (cytochrome *bc*1 complex), complex IV (cytochrome *c* oxidase) and complex V (ATP synthase), and all are localized on the inner mitochondrial membrane. During electron transport, complexes I, III and IV pump protons from the mitochondrial matrix to the inter-membrane space, resulting in an increase in the membrane potential across the inner mitochondrial membrane. Following this, complex V actively allows the flow of protons back to the matrix, resulting in the generation of energy in the form of ATP from adenosine diphosphate (26). The OXPHOS system consists of 85 subunits as components of various complexes, in which 13 are encoded in mtDNA. These 13 mitochondrial-encoded proteins constitute various subunits that make up four OXPHOS complexes (complex I, III, IV and V) (27). Studies have demonstrated that the mtDNA mutations alter the mitochondrial-encoded subunits and play a significant role in numerous malignancies, including renal oncocytoma, thyroid oncocytic carcinoma, bladder, prostate and colon cancer (28–32).

MtDNA mutations have also been identified in HCC (33–35). These mutations potentially cause the defects of mitochondrial-encoded subunits in the OXPHOS system and result in mitochondrial dysfunction in HCC (36). It has been demonstrated that the defects of complexes III and IV can be detected by immunohistochemical (IHC) staining in normal human and cirrhotic liver during aging. However, these defects were not shown in 27 sections of HCC tissues (37). The actual defects of mitochondrial-encoded subunits in HCC and the association with the outcome remain unclear. The present study aimed to examine the expression of the defects of the mitochondrial-encoded OXPHOS subunits; complex I subunit 6 (CI-6), complex III subunit 3 (CIII-3), complex IV subunit 1 (CIV-1) and complex V subunit 6 (CV-6), in surgically removed HCCs, and their correlations with clinicopathological factors and prognosis.

## Patients and methods

### Patients and specimens

Under the approval of the Institutional Review Board, Chang Gung Medical Center (Taoyuan, Taiwan), 102 human HCC tissues with a clear surgical margin were randomly collected from patients undergoing curative resection at Chang Gung Memorial Hospital at Linkou (Taoyuan, Taiwan) between 1996 and 2006 and stored in the Chang Gung tissue bank. The tissues were retrieved from the tissue bank and formalin-fixed and paraffin-embedded for the IHC study. The inclusion criteria included pathological diagnosis of HCC, no anticancer therapy prior to the surgery, curative resection, adequately formalin-fixed and paraffin-embedded tissues, complete clinicopathological data, regular follow-up and reliable medical records. The exclusion criteria included pregnancy, other co-existing malignancies and mortality due to unrelated diseases.

The clinical parameters included age, gender, chronic HBV, chronic HCV, liver cirrhosis, ascites, α-fetoprotein (AFP), albumin, bilirubin, prothrombin time, creatinine, aspartate aminotransferase (AST), alanine aminotranferease (ALT), alcohol use (average alcohol consumption of >210 g per week in males or >140 per week in females over at least a 2-year period), local recurrence, time to local recurrence, distant metastasis, time to distant metastasis, mortality and overall survival time. The pathological findings, including tumor grade, microvascular invasion, macrovascular invasion, capsule, tumor number and largest tumor diameter, were examined by two experienced pathologists without information of the clinical data.

Curative resection was defined as a complete resection of all tumors with the margin free from cancer invasion by histological examination, no tumor thrombus in the main trunk, two major portal branches, hepatic veins or bile duct and no extrahepatic metastasis (38). HCC diagnosis and grading were established according to World Health Organization criteria (39). Macrovascular invasion was defined as the invasion of the tumor into the vessels that can be identified during macroscopic examination or radiological imaging. The definition of microvascular invasion included: i) Presence of tumor cells forming a plug or polyp in a subendothelial location, partially or totally covered by endothelial cells; ii) presence of tumor thrombus, partially or totally covered by the endothelium; iii) vascular structures involved can be portal vein branches, hepatic vein branches or capsule vessels, inside the tumor or closely situated to the tumor edge; and iv) invasion of arteries and lymphatic vessels (40). Local recurrence was defined as intrahepatic recurrence and distant metastasis was equal to extrahepatic metastasis.

### Ethics statement

This study was approved by Chang Gung Medical Foundation Institutional Review Board (no. 100-1728B, between 01/08/2011 and 31/07/2014; Taoyuan, Taiwan). The Institutional Review Board waived the requirement for informed consent from the participants as the present study was a retrospectively observational analysis and the information identifying patients was not included in the collected data. Tissue samples were obtained from the Tissue Bank of Linkou Chang Gung Memorial Hospital (Taoyuan, Taiwan) through approval of the committee.

### IHC analysis

The IHC stains were performed for detection of the expression defects of CI-6, CIII-3, CIV-1 and CV-6 with rabbit anti-nicotinamide adenine dinucleotide (NADH) dehydrogenase subunit 6 polyclonal antibody (Abcam, Cambridge, MA, USA), goat anti-cytochrome *b* polyclonal antibody (Santa Cruz Biotechnology, Inc., Santa Cruz, CA, USA), mouse anti-OXPHOS complex IV subunit I monoclonal antibody (Invitrogen Life Technologies, Carlsbad, CA, USA) and rabbit anti-mt-ATP6 polyclonal antibody (Abcam), respectively. Deparaffinized rehydrated sections were treated with H_2_O_2_ (3% in distilled water) for 15 min, pre-incubated with normal serum (goat serum for rabbit anti-NADH dehydrogenase subunit 6 antibody and anti-mt-ATP6 antibody; horse serum for anti-OXPHOS complex IV subunit I monoclonal antibody; and rabbit serum for anti-cytochrome *b*) with phosphate-buffered saline in the proportion of 1:10 for 40 min. Following this, the sections were then incubated with the specific primary antibody (1 h at 37°C for anti-NADH dehydrogenase subunit 6 antibody and anti-OXPHOS complex IV subunit I monoclonal antibody; and overnight at 4°C for anti-cytochrome *b* and anti-mt-ATP6 antibody) and the secondary antibody of the VECTASTIN Elite ABC kit [anti-rabbit immunoglobulin G (IgG) for rabbit anti-NADH dehydrogenase subunit 6 antibody and anti-mt-ATP6 antibody; anti-mouse IgG for anti-OXPHOS complex IV subunit I monoclonal antibody; and anti-goat IgG for anti-cytochrome *b*; Vector Laboratories, Inc., Burlingame, CA, USA] for 40 min at room temperature. Visualization was performed with the 3,3′-diaminobenzidine substrate kit, SK-4100 (Vector Laboratories, Inc.). The stained sections were examined separately by two experienced pathologists, who were blinded to the clinical information. The defect areas (absence of reactivity) in the tumor were estimated and recorded as percentages. If there was a discrepancy in the interpretation, a consensus was reached between the two pathologists by reviewing slides simultaneously. Tissues were classified into two groups based on the percentages of the defect areas in a single cross-section of the HCC samples (major defect, >25% HCC area; and minor defect, ≤25% of the HCC area) for further evaluation.

### Statistical analysis

Numerical data are presented as mean ± standard deviation, while categorical data were expressed as absolute number and percentages. The χ^2^ test was used for group comparisons involving binary data and independent samples. Mann-Whitney U test was used for the largest tumor size, AFP, AST, ALT, local recurrent time, distal metastatic time and disease-free survival time, due to a skewed distribution. Other numerical data were evaluated by Student’s t-test. The results were considered to indicate a statistically significant difference when P<0.05. Univariate and multivariate analyses were performed by Cox proportional hazards regression to identify independent risk factors for mortality. The results were presented with hazard ratio (HR), 95% confidence interval (CI) and P*-*value. Survival curves were also analyzed by the Kaplan-Meier curve and log-rank test. All statistical calculations were performed using SPSS, 18.0 software (SPSS, Inc., Chicago, IL, USA).

## Results

### Patient characteristics

The age of the patients at the time of surgery ranged between 25 and 89 years, and the male to female ratio was 3.64. The tumor-node-metastasis stage was between stages I and IIIA, according to the American Joint Committee on Cancer Cancer Staging Manual, seventh edition (2010) (41). The cohort of the present study included 51 liver cirrhosis patients, 38 alcohol users, 70 chronic HBV carriers, 17 chronic HCV carriers and 3 HBV and HCV co-infection patients. The average follow-up duration was 50.29±43.50 months. The rates of mortality, local recurrence, distal metastasis, 5-year survival and 5-year disease-free survival were 36.27, 64.71, 39.22, 37.25 and 22.55%, respectively.

### Clinicopathological characteristics

**Figure 1:**
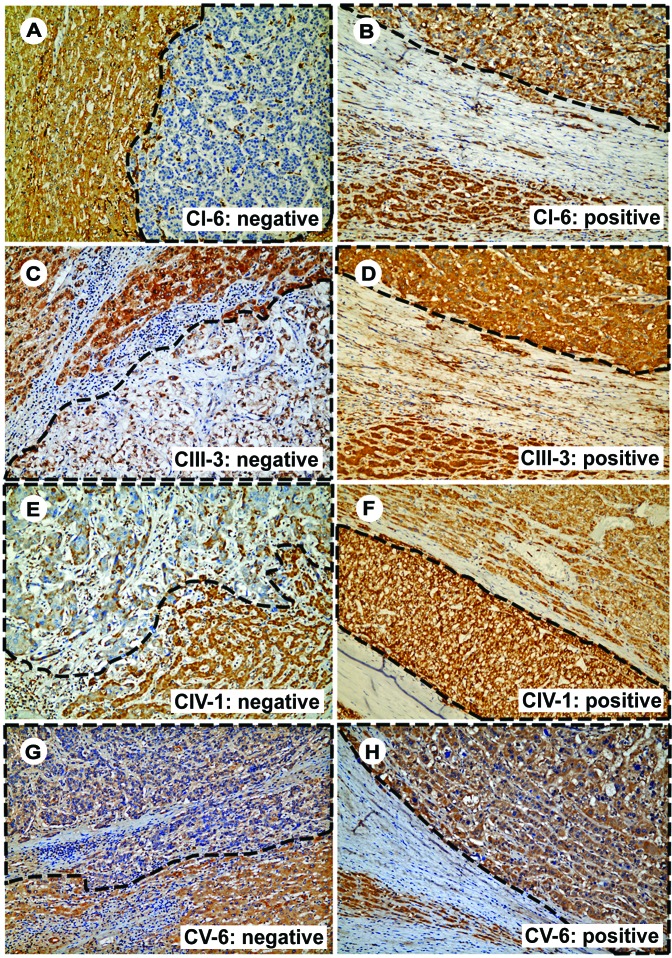
Immunohistochemical staining of (A and B) complex I subunit 6, (C and D) complex III subunit 3, (E and F) complex IV subunit 1 and (G and H) complex V subunit 6 in hepatocellular carcinoma, within the dotted line.

The majority of the HCC tissues contained various degrees of mitochondrial-encoded OXPHOS enzyme defects. The expression defects of CI-6, CIII-3, CIV-1 and CV-6 were present in 100, 98, 98 and 93% of the tissues assessed, respectively. The major and minor defects were defined as IHC staining defect area of >25% and ≤25%, respectively (Fig. 1). According to this definition, the frequency of the major defect for CI-6, CIV-1, CIII-3 and CV-6 was 67.7, 50.0, 31.3 and 24.5%, respectively. The CI-6 major-defect group had lower ALT levels compared with the minor-defect group [35 (10-280) and 59 (9-371) U/l, P=0.038]. The CIII-3 major-defect group had fewer encapsulated tumors (56.25 and 75.71%, P=0.047) and higher albumin levels (4.03±0.44 and 3.67±0.68 g/dl, P=0.002), compared with the minor-defect group. The CIV-1 major-defect group had higher AFP levels [204.08 (0.9-89637.7) and 21.92 (2-286980) ng/ml, P=0.021] and higher AST levels [63 (12-351) and 36 (11-278) U/l, P=0.018] compared with the minor-defect group. The CV-6 major-defect group was older (63.12±9.80 and 53.86±15.53 years, P=0.001) compared with the minor-defect group. There was no other significant difference in clinicopathological parameters between the major- and minor-defect groups (Table I).

### Clinicopathological parameters and OXPHOS expression defects associated with post-operative survival in HCC

**Figure 2:**
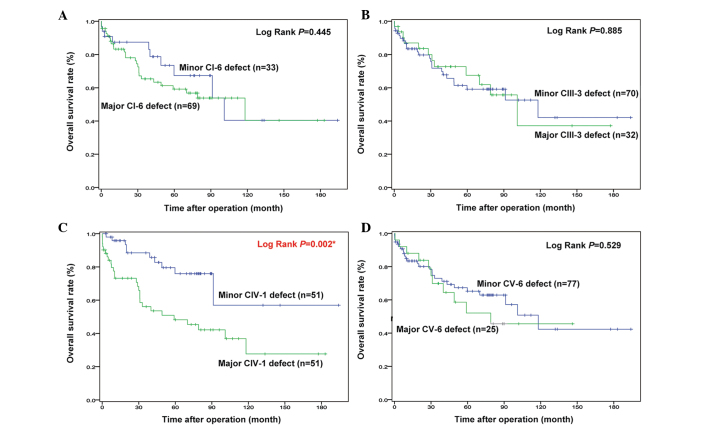
Overall survival curves of hepatocellular carcinoma with (A) complex I subunit 6 (CI-6), (B) complex III subunit 3 (CIII-3), (C) complex IV subunit 1 (CIV-1) and (D) complex V subunit 6 (CV-6) defects, assessed by Kaplan-Meier survival estimates and the log-rank test.

Univariate analysis revealed that ascites (HR, 6.016; 95% CI, 2.364–15.309; P<0.001), albumin levels (HR, 0.524; 95% CI, 0.304–0.905; P=0.020), time to local recurrence (HR, 0.948; 95% CI, 0.927–0.970, P<0.001), time to distant metastasis (HR, 0.934; 95% CI, 0.915–0.953; P<0.001), disease-free survival time (HR, 0.948; 95% CI, 0.927–0.969; P<0.001) and major CIV-1 defect (HR, 3.050; 95% CI, 1.471–6.324; P=0.003) were associated with the overall survival time (Table II). These six factors were further analyzed by multivariate analysis. It was found that the major CIV-1 defect (HR, 5.676; 95% CI, 2.243–14.360; P<0.001) and the time to distant metastasis (HR, 0.924; 95% CI, 0.894–0.955; P<0.001) were significant independent predictive factors for overall survival time (Table III). Kaplan-Meier survival analysis using the log-rank test (Fig. 2) also showed that the CIV-1 major-defect group had an unfavorable survival time compared with that of the minor-defect group (P=0.002). Additionally, the major CI-6 and CV-6 defect groups appeared to have a poor survival rate within 8 years, but it did not reach statistical significance. There was no significant difference between major- and minor-defect groups of CIII-3, according to the Kaplan-Meier survival curve.

## Discussion

According to the present study, the majority of the HCC tissues contained various degrees of mitochondrial-encoded OXPHOS complex defects, indicating the impairment of ATP production by pyruvate oxidation in mitochondria. This result is compatible with the phenomenon of the Warburg effect, indicating that energy is mainly supplied by glycolysis in cancer cells (19). To understand whether the degree of OXPHOS defects correlate with clinicopathological presentation and prognosis, the major and minor defects were defined as IHC staining defect area of >25% (major) and ≤25% (minor). The frequencies of the major defect were as follows: CI-6, 67.65%; CIV-1, 50.00%; CIII-3, 31.37%; and CV-6, 24.51%). These results indicate that HCC tissues tend to have a larger portion of CI-6 and CIV-1 defects, rather than CIII-3 and CV-6. It has been indicated that the OXPHOS enzyme defects are associated with aging (37). However, in the present study, only the major CV-6 defect was correlated with age (63.12±9.80 and 53.86±15.53 years, P=0.001), indicating that other mechanisms are responsible for the generation of these defects in HCC, rather than aging. Through univariate and multivariate analyses, the major CIV-1 defect was found to be a negative predictive factor for HCC patients following curative resection.

Complex IV is the terminal oxidase of the respiratory chain in the mitochondria. In mammals, it contains 13 subunits, of which 3 catalytic subunits (subunit 1, 2 and 3) are encoded by the mitochondrial genes. The remaining 10 subunits are encoded by nuclear DNA and are suspected to be involved in the regulation and/or assembly of the complex (41). Complex IV represents the rate-limiting enzyme of the mitochondrial respiratory chain and its activity is an indicator of the oxidative capacity of the cells. It is therefore fated to be a central site of regulation of oxidative phosphorylation, proton pumping efficiency, ATP and reactive oxygen species production, which in turn affect cell signaling and survival (42,43). Complex IV can also be incorporated into larger structures containing complex I, II and III, and the mobile electron carriers, cytochrome *c* and ubiquinol, to form functional supercomplexes; respirasomes (44,45). These supercomplexes may stabilize the individual complexes (46) to enhance respiration due to coordinated channeling of electrons (47). Due to the significance of complex IV, organisms have evolved various levels of regulation for its activity. A defect of complex IV has been proved to result in numerous diseases, including Leber hereditary optic neuropathy, Leigh syndrome, recurrent myoglobinuria mitochondrial disorder, deafness sensorineural mitochondrial disorder and colorectal cancer (48–54). However, their roles in HCC are not clear. The present study firstly revealed the potential significance of the CIV-I defects in HCC. Further experiments involving knocking out the CIV-I gene by small hairpin RNA in the Huh7 HCC cell line will be performed to demonstrate their effects on liver tumor.

It has been demonstrated that OXPHOS protein defects can be found in normal and cirrhotic human liver. A study by Müller-Höcker *et al* (37) enrolled 107 normal livers (including 11 HCC cases) and 64 cirrhotic livers (including 16 HCC cases) and aimed to detect the respiratory chain protein (complex II, III and IV) and complex V defects in normal and cirrhotic liver during aging. Enzyme histochemistry was performed to detect complex II, IV and V, and immunohistochemistry with polyclonal antibody (against both nuclear and mitochondrial subunits) was conducted for detection of total complex III and complex IV subunits 2, 3 and 4. In normal livers, the respiratory chain defects were detected in 57% cases, and 87% in advanced age (>50 years old). In cirrhotic liver, the overall frequency of defects was higher (78%) compared with normal liver, but was inverse (60%) in advanced age. However, there were no defects of complex II, III, IV and V observed in 27 HCC tissues in their study. On the contrary, the present study showed that the majority of resected HCC tissues contain various degrees of mitochondrial-encoded subunits defects. The conflicting results may be due to the varied HCC tissue origins (metastatic and primary), various OXPHOS enzymes examined and different methods performed. Nevertheless, the results of the present study firstly demonstrated that the majority of HCCs contain mitochondrial-encoded subunit defects, indicating a potential role of these defects in hepatocarcinogenesis.

MtDNA mutations have been detected in HCC. A study by Lee *et al* (33) examined the D-loop mutations and mtDNA numbers in 61 HCCs and the corresponding non-tumor sections. The results showed that 39.3% of HCCs carried somatic mutations in the D-loop of mtDNA. A significant decrease in the copy number of mtDNA was also detected in 60.5% of patients with HCC (33). A small-scale study examining 18 HCC patients also demonstrated a significant decrease of mtDNA copy number, particularly in females but not in males with HCC (34). Another study examined 44 HCCs and in total 13 somatic mtDNA mutations were found in 11 HCC samples (35). Among these mutations, the T6768C (CIV-1), G7976A (complex IV subunit II), G9267 (complex IV subunit 3) and A111708 (complex I subunit 4) mutations could result in amino acid substitutions in the highly conserved regions and have the potential to cause mitochondrial dysfunction in HCC. Although the mutation of mtDNA was not examined in the present study, the mitochondrial-encoded OXPHOS subunit defects shown may have at least partially resulted from mtDNA mutations and the decrease of mtDNA copy number.

There were limitations in the present study regarding the case number, the choice of specimens and the lack of western blotting, reverse transcription polymerase chain reaction and mtDNA analysis. In total, 102 HCC tissues were collected with an average follow-up duration of 50.29 months (range, 0.07–194 months). The case number appeared to not be enough for subgroup analyses. Due to the strength of the IHC staining, it was more difficult to evaluate objectively and it was not associated with prognosis (data not shown); the percentages of IHC stain defect areas were chosen in a single cross-section of HCC without significant necrosis to evaluate the degree of the OXPHOS complex defects. The same scoring system read by the pathologists is also widely used in clinical practice, including HER2 in breast cancer and Ki-67 in neuroendocrine tumor (55,56). Although it was easy for clinical applications, it may have bias between different pathologists when scoring the defects areas. We hypothesize that this bias can be reduced by repeated reading and experienced examiners.

To the best of our knowledge, the present study was the first to demonstrate the correlation between OXPHOS subunit defects and overall survival time in HCC patients. The results showed that the majority of HCCs contained mitochondrial-encoded OXPHOS subunit defects. Among these, the major CIV-1 defect was a negative predictor for post-operative survival in HCC. It may provide a simple way to predict the outcome in this group of patients.

## Figures and Tables

**Table I:** Correlation between CI-6, CIII-3, CIV-1 and CV-6 and clinicopathological characteristics in 102 human HCC tissues.

	CI-6 defect			CIII-3 defect			CIV-1 defect			CV-6 defect		
				
Parameter	Minor	Major	P-value	Minor	Major	P-value	Minor	Major	P-value	Minor	Major	P-value
Case number, n (%)	33 (32.35)	69 (67.65)		70 (68.63)	32 (31.37)		51 (50)	51 (50)		77 (75.49)	25 (24.51)	
Gender, n (M/F)	23/10	57/12	0.138	53/17	27/5	0.324	42/9	38/13	0.336	58/19	22/3	0.181
Age, years	58.48±14.22	55.00±15.10	0.269	57.90±15.64	52.25±12.27	0.052	55.63±16.30	56.63±13.37	0.736	53.86±15.53	63.12±9.80	0.001^a^
HBsAg, n (%)	21 (63.64)	52 (75.36)	0.219	47 (67.14)	26 (81.25)	0.143	35 (68.63)	38 (74.51)	0.510	57 (74.03)	16 (64.00)	0.334
HCV, n (%)	9 (27.27)	11 (15.94)	0.178	15 (21.43)	5 (15.63)	0.493	7 (13.73)	13 (25.49)	0.135	14 (18.18)	6 (24.00)	0.524
Liver cirrhosis, n (%)	16 (48.48)	35 (50.72)	0.832	37 (52.86)	14 (43.75)	0.393	23 (45.10)	28 (54.90)	0.322	37 (48.05)	14 (56.00)	0.490
Tumor grade	2.50±0.70	2.42±0.61	0.547	2.47±0.66	2.38±0.60	0.535	2.48±0.66	2.41±0.62	0.582	2.47±0.62	2.36±0.70	0.449
Microvascular invasion, n (%)	12 (36.36)	25 (36.23)	0.990	23 (32.86)	14 (43.75)	0.288	19 (37.25)	18 (35.29)	0.837	32 (41.56)	5 (20.00)	0.051
Macrovascular invasion, n (%)	4 (12.12)	3 (4.35)	0.146	5 (7.14)	2 (6.25)	0.869	1 (1.96)	6 (11.76)	0.050	7 (9.09)	0 (0.00)	0.118
Capsule, n (%)	25 (75.76)	46 (66.67)	0.350	53 (75.71)	18(56.25)	0.047^a^	33 (64.71)	38 (74.51)	0.282	53 (68.83)	18 (72.00)	0.765
Tumor number	1.58±0.97	1.59±0.85	0.922	1.46±0.74	1.88±1.10	0.057	1.55±0.86	1.63±0.92	0.656	1.55±0.82	1.72±1.06	0.393
Largest tumor size (diameter, cm)	5.3 (2–20)	5 (1–77.5)	0.429	5 (1–20)	6.5 (2–77.5)	0.323	4.8 (1.8–20.5)	6 (1–77.5)	0.254	5.5 (2–20.5)	4 (1–77.5)	0.190
Ascites, n (%)	3 (9.09)	6 (8.70)	0.911	8 (11.43)	1 (3.13)	0.165	3 (5.88)	6 (11.76)	0.309	7 (9.09)	2 (8.00)	0.854
AFP, ng/ml	38 (2-44890.2)	59 (0.9-286980)	0.468	35.5 (1.47-286980)	133.5 (0.9-89637.7)	0.070	21.92 (2-286980)	204.08 (0.9-89637.7)	0.021^a^	50.22 (1.47-286980)	61 (0.9-4585)	0.531
Albumin, g/dl	3.70±0.63	3.83±0.64	0.356	3.67±0.68	4.03±0.44	0.002^a^	3.87±0.62	3.70±0.65	0.201	3.76±0.67	3.85±0.50	0.564
Billirubin, mg/dl	0.98±0.48	1.04±0.92	0.736	1.10±0.92	0.85±0.38	0.153	0.91±0.42	1.13±1.04	0.170	1.04±0.90	0.98±0.36	0.750
Prothrombin time, sec	12.4±1.13	12.55±1.67	0.651	12.65±1.67	12.15±1.01	0.125	12.44±1.78	12.55±1.20	0.722	12.61±1.61	12.18±1.13	0.220
Creatinine, mg/dl	1.22±0.76	1.25±1.26	0.911	1.24±0.95	1.25±1.45	0.963	1.15±0.61	1.325±1.47	0.439	1.10±0.52	1.68±2.04	0.172
AST, U/l	52 (11-278)	36 (12-351)	0.520	40.5 (11-351)	39 (15-312)	0.908	36 (11-278)	63 (12-351)	0.018^a^	45 (11-351)	35 (12-260)	0.361
ALT, U/l	59 (9-371)	35 (10-280)	0.038^a^	41.5 (9-371)	41.5 (13-280)	0.905	40 (9-371)	47 (13-280)	0.322	45 (9-371)	33 (13-279)	0.800
Alcohol use, n (%)	14 (42.42)	24 (34.78)	0.455	26 (37.14)	12 (37.50)	0.972	15 (29.41)	23 (45.10)	0.101	28 (36.36)	10 (40.00)	0.744

a P<0.05. Largest tumor size, AFP, AST and ALT are presented by median (range). The remaining data are presented as the mean ± standard deviation, unless stated otherwise. AFP, α-fetoprotein; AST, aspartate aminotransferase; ALT, alanine aminotranferase; CI-6, complex I subunit 6; CIII-3, complex III subunit 3; CIV-1, complex IV subunit 1; CV-6, complex V subunit 6; F, female; HCC, hepatocellular carcinoma; HCV, hepatitis C virus; M, male; minor, immunohistochemical-negative area ≤25%; major, immunohistochemical-negative area >25%.

**Table II:** Univariate analysis of parameters associated with overall survival time.

Parameter	Patient data	SE	HR	95% CI	P-value
Gender, male	80 (78.4%)^a^	0.384	0.964	0.454~2.046	0.924
Age, years	56.13±14.84	0.011	1.012	0.990–1.034	0.292
HBsAg	73 (71.6%)^a^	0.391	0.999	0.464~2.150	0.999
HCV	20 (19.6%)^a^	0.485	0.727	0.281~1.879	0.511
Liver cirrhosis	51 (50.0%)^a^	0.332	0.919	0.479~1.762	0.798
Tumor grade	2.44±0.64	0.255	1.001	0.608–1.649	0.996
Microvascular invasion	37 (36.3%)^a^	0.366	1.181	0.577~2.418	0.649
Macrovascular invasion	7 (6.9%)^a^	0.535	1.799	0.630~5.139	0.273
Capsule	71 (69.6%)^a^	0.386	1.272	0.597~2.709	0.533
Tumor number	1.59±0.88	0.181	1.136	0.797–1.619	0.481
Largest tumor size (diameter, cm)	5.1 (1.0–77.5)^b^	0.012	1.012	0.989–1.037	0.313
Ascites	9 (8.8%)^a^	0.477	6.016	2.364~15.309	<0.001^c^
AFP (ng/ml)	50.26 (0.9-286980)^b^	0.000	1.000	1.000~1.000	0.585
Albumin (g/dl)	3.78±0.64	0.278	0.524	0.304–0.905	0.020^c^
Billirubin (mg/d)	1.02±0.80	0.219	1.163	0.758–1.785	0.490
Prothrombin time (sec)	12.50±1.51	0.083	1.159	0.985–1.365	0.076
Creatinine (mg/dl)	1.24±1.12	0.111	1.171	0.942–1.456	0.155
AST (U/l)	39.5 (11-351)^b^	0.002	1.004	1.000–1.009	0.073
ALT (U/l)	41.5 (9-371)^b^	0.003	0.999	0.993~1.005	0.692
Alcohol use	38 (37.3%)^a^	0.332	1.387	0.723~2.660	0.325
Local recurrent time (month)	14 (0.07-194)^b^	0.011	0.948	0.927–0.970	<0.001^c^
Distal metastatic time (month)	34.5 (0.07–194)^b^	0.010	0.934	0.915–0.953	<0.001^c^
Disease free survival time (month)	14 (0.07–194)^b^	0.011	0.948	0.927–0.969	<0.001^c^
Major complex I subunit 6 defect	69 (67.6%)^a^	0.371	1.326	0.641–2.742	0.446
Major complex III subunit 3 defect	32 (31.4%)^a^	0.352	0.950	0.477–1.893	0.885
Major complex IV subunit 1 defect	51 (50.0%)^a^	0.372	3.050	1.471~6.324	0.003^c^
Major complex V subunit 6 defect	25 (24.5%)^a^	0.361	1.255	0.618–2.546	0.530

a Number (%);

b median (range);

c P<0.05.

Data are presented as the mean ± standard deviation, unless stated otherwise. SE, standard error; HR, hazard ratio; CI, confidence interval; AFP, α-fetoprotein; AST, aspartate aminotransferase; ALT, alanine aminotranferase; HCV, hepatitis C virus; major, immunohistochemical-negative area >25%.

**Table III:** Multivariate analysis of parameters associated with overall survival time.

Parameter	SE	HR	95% CI	P-value
Ascites	0.500	1.697	0.637~4.521	0.290
Albumin, g/dl	0.341	1.012	0.519~1.975	0.971
Local recurrent time, month	0.607	0.791	0.241~2.598	0.699
Distal metastatic time, month	0.017	0.924	0.894~0.955	<0.001^a^
Disease-free survival time, month	0.609	1.262	0.383~4.159	0.702
Major complex IV subunit 1 defect	0.474	5.676	2.243~14.360	<0.001^a^

a P<0.05.

SE, standard error; HR, hazard ratio; CI, confidence interval; major, immunohistochemical-negative area >25%.
